# First person – Anne Cathrine Hyde

**DOI:** 10.1242/bio.062594

**Published:** 2026-04-24

## Abstract

First Person is a series of interviews with the first authors of a selection of papers published in Biology Open, helping researchers promote themselves alongside their papers. Anne Cathrine Hyde is first author on ‘ Assessing frailty in aged zebrafish using a quick pseudo-frailty index’, published in BiO. Anne Cathrine conducted the research described in this article while a PhD candidate in Fredericus van Eeden's lab at The University of Sheffield, Sheffield, UK. She is now a postdoctoral research associate in the lab of Hilary Ashe at The University of Manchester, Manchester, UK, investigating how RNA molecules affect development and cellular processes.

**Figure:**
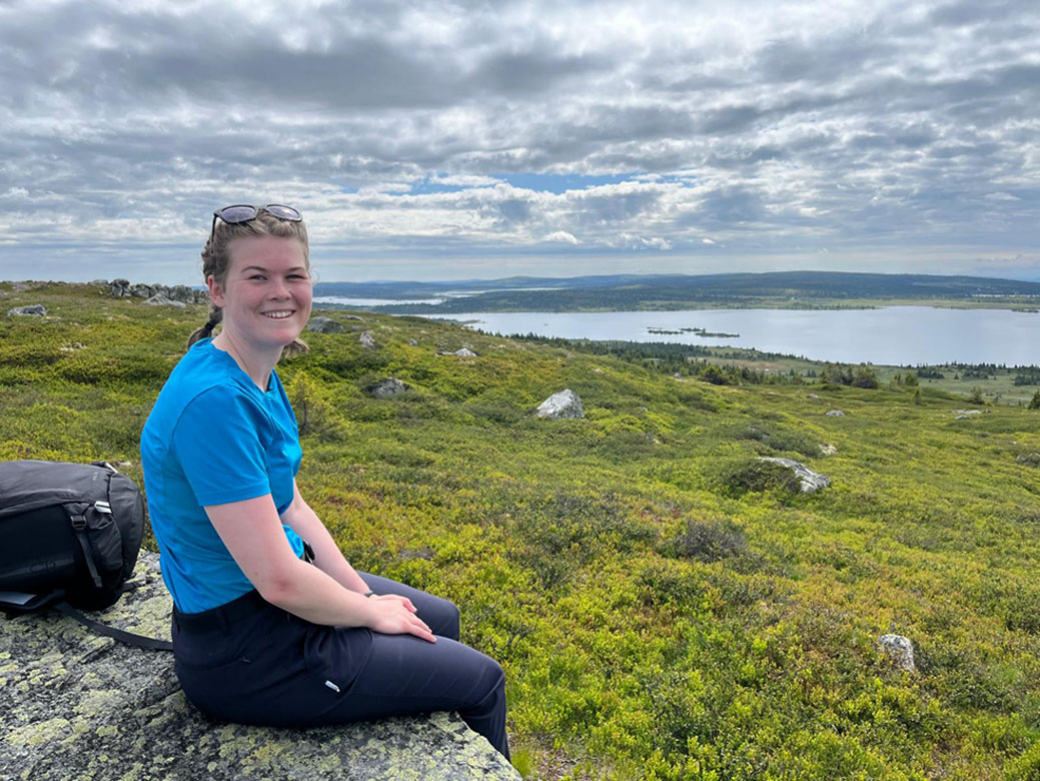
Anne Cathrine Hyde

**Describe your scientific journey and your current research focus**

From year 8 in school, I have been focused on a career in biological research, with a dream to study genetics. I have a very happy memory of doing my oral exam in genetics in year 10, where I extracted DNA from raspberries and tried to explain Punnett squares, failing miserably at both, but it didn't put me off. My dreams made me study higher-level biology in my International Baccalaureate, when my biology teacher Dr Joanne Haugaasen inspired me to move from Norway to England to study biological sciences at the University of East Anglia. In Norwich, my fondness for developmental biology grew when I was introduced, during my Master's degree, to Professor Grant Wheeler's lab, who studied neural crest development in *Xenopus* embryos, with a focus on enhancer regulation of neural crest genes. From this moment, I was certain I wanted a career in research, so I continued onto a PhD at The University of Sheffield at the start of COVID in 2021. Struggling to compete for the PhDs in developmental biology, I decided to go out of my comfort zone and do a PhD with the Healthy Lifespan Institute in Dr Fredericus van Eeden and Professor Sherif El-Khamisy's labs, studying the role of ribonucleotide insertion into genomic DNA during ageing in zebrafish. During the PhD, I got to continue my interests in genetics by making several CRISPR lines and playing around with DNA damage. However, while studying ageing was fun and rewarding, I missed having a focus on developmental biology. Therefore, I was very lucky to get a job as a postdoctoral researcher in Professor Hilary Ashe's lab at The University of Manchester to study mRNA degradation during development in the *Drosophila* embryo, which is where I am currently working, and where I can continue my interest in genetics and RNA biology.

**Who or what inspired you to become a scientist?**

I have always been a curious person, a characteristic that has been encouraged by my family who have always inspired me. My family is a very academic family, and both my parents have Master's degrees, with my mother having a Master's degree in plant biology and my grandmother having a PhD in health sciences and ageing. Therefore, I knew that biology, university and, ultimately, a PhD was always the way I wanted to go. Further, Dr Joanna Haugaasen, my biology teacher for the International Baccalaureate was very encouraging during my extended essay researching the effect of copper pollution on plant development.

**How would you explain the main finding of your paper?**

Ageing is a process most of us will go through. However, studying human ageing can be challenging due to how individual we all are. Therefore, using model organisms is a good starting point to understand the basics of ageing, then to later be able to apply the findings to humans. Zebrafish are good models for ageing as they start ageing between 2.5 and 3 years, they share many ageing features with humans, and we can house them similarity throughout their lives, reducing variability. However, like humans, the fish do not all age in the same way; some fish will become sicker and more frail due to their ageing than others, which can skew research. Therefore, here, we have developed a scoring system to determine which zebrafish are more frail than other fish to be able to separate out the different types of ageing. The aim is to make a common way for researchers to assess frailty within zebrafish to be more able to understand ageing.

… the frailty scoring system that I had made for my zebrafish correlated with what the experts within zebrafish biology and zebrafish ageing thought of frailty …

**What are the potential implications of this finding for your field of research?**

The aim of this paper is to create a common way for researchers to assess frailty within zebrafish. This way, we can all better understand the ageing process together. We are not claiming that the way in the paper is the best way to assess frailty, however, we would like there to potentially be a discussion so that all zebrafish ageing research can evaluate the same fish.

**Figure:**
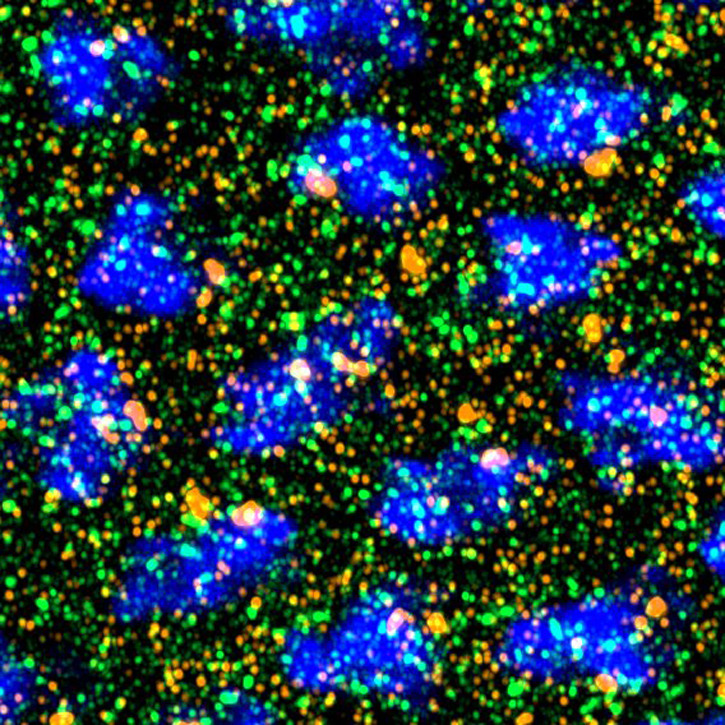
Z projection of cv-2 mRNA (single-molecule fluorescence *in situ* hybridization; yellow) in relation to P bodies marked by an anti-Me31B antibody (green) in a nuclear cycle 14 *Drosophila melanogaster* embryo (DAPI, blue).

**Which part of this research project was the most rewarding?**

The most rewarding part of this research project was to see that the frailty scoring system that I had made for my zebrafish correlated with what the experts within zebrafish biology and zebrafish ageing thought of frailty, confirming that the five criteria I have chosen as the best and quickest markers for frailty were good markers.

**What do you enjoy most about being an early-career researcher?**

The best thing for me as an early-career researcher is when something I have worked on for a long time works. Especially if that thing is something that hasn't worked on multiple occasions and I have finally figured out how to get it to work. The feeling I am left with, knowing I don't need to work on that one thing again, is the greatest feeling I know of.

Don't just find a topic in your undergraduate and stick with that one topic; it limits your possibilities in the future and narrows your skill sets

**What piece of advice would you give to the next generation of researchers?**

Expand your knowledge. Don't just find a topic in your undergraduate and stick with that one topic; it limits your possibilities in the future and narrows your skill sets. The best decision I made was to go into ageing, even though I'm back at development now.

**What's next for you?**

I am doing at least another 2 years as a postdoc in Hilary's lab. After that, I am open to a lot of different things. I'm not sure if I want to stay in academia; maybe I'll venture into industry, or some form of teaching.
